# Hepatic Diffuse Large B-cell Lymphoma: A Case Report and Review of Literature of Primary Liver Tumors

**DOI:** 10.7759/cureus.54947

**Published:** 2024-02-26

**Authors:** Manuel A Pérez-Turrent, Jesús I Hernández-Solís, Estrella Elizabeth Sánchez Antonio, Cristina V Trinidad-Esparza, José J Herrera-Esquivel

**Affiliations:** 1 Gastrointestinal Endoscopy, Hospital General "Dr. Manuel Gea González", Mexico City, MEX; 2 Pathological Anatomy, Instituto Nacional de Cancerología, Mexico City, MEX

**Keywords:** us-guided fine needle aspiration, endoscopic ultrasound, primary liver tumors, malignant liver tumors, diffuse large b-cell hepatic lymphoma

## Abstract

Liver tumors rank as the fourth most common cause of cancer. This case report highlights a 45-year-old female patient who presented persistent abdominal pain and no other symptoms. Initially, she was approached with a probable hepatitis of unknown origin, but her condition worsened rapidly. An endoscopic ultrasound was used to characterize the lesion, and a fine needle biopsy of the lesion was performed which revealed a diffuse large B-cell lymphoma that is CD20+ and Ki67+. Hepatic diffuse large B-cell lymphoma, as diagnosed in the patient, is a rare type of lymphoma that arises in the liver. The treatment usually involves chemotherapy, immunotherapy, and radiation therapy. However, the prognosis depends on the stage of the disease and the patient's overall health. This case reinforces the importance of considering hepatic diffuse large B-cell lymphoma in differential diagnosis for primary liver neoplasia.

## Introduction

Liver tumors are the fourth most common cause of cancer after lung, colorectal, and stomach cancers [1]. Malignant liver tumors can be separated into two groups: primary tumors which originate in the liver and secondary tumors which are caused by dissemination from a primary neoplasia.

Lymphoma is a form of cancer that occurs when B or T lymphocytes proliferate unregulated and display immortality [2]. Hepatic lymphomas are classified into two forms: The first group is called primary hepatic lymphomas (PHL). These lymphomas do not affect the spleen, lymph nodes, bone marrow, or other lymphatic structures [3]. They are considered extremely rare and represent only 0.016% of all non-Hodgkin lymphoma cases. Only 0.4% occur in the liver, and up to five years ago, only 300 cases had been reported in the literature [4]. The etiology of PHL is unknown; it presents on average at 50 years of age and develops more often in males [3,4]. The other group, called secondary hepatic lymphomas, is considered more common, as approximately 40% of patients with systemic non-Hodgkin lymphoma have liver involvement [5]. In this article, we present a case report and a literature review of primary liver tumors.

## Case presentation

**Figure 1:**
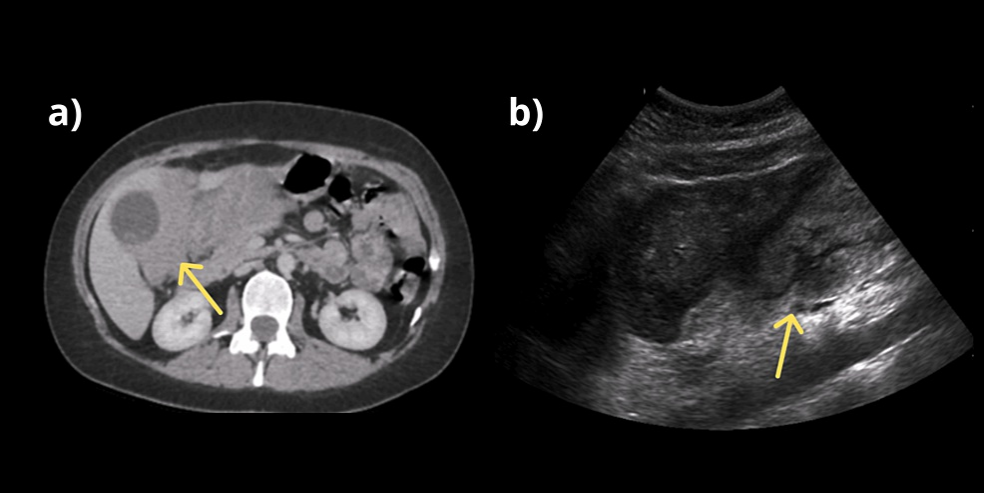
Initial scans on admission a) Initial CT scan showing a hypodense mass involving both liver lobes. b) Liver ultrasound showing a large, isoechoic mass

We present a case of a 45-year-old female initially evaluated for abdominal pain in the right hypochondrium and umbilical region persistent for two months. Past medical history and family history although studied were not relevant to the current disease. She was attended to by a general practitioner who made an inaccurate diagnosis of an unknown origin hepatitis without proper studies or further approach. Her symptoms rapidly progressed and worsened. Physical examination at the moment of her admission demonstrated right upper quadrant pain and dull percussion in the right costal margin. Axillary, cervical, and inguinal lymph node chains were not palpable, and there were no signs of adenopathy. Initial abdominal CT showed a solid lesion involving the liver, stomach, pancreas, duodenum, left adrenal gland, and gallbladder, with dilation of the bile duct and free fluid (Figure 1).

**Figure 2:**
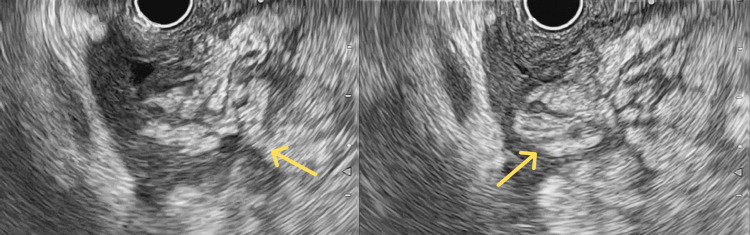
Endoscopic ultrasound Endoscopic ultrasound showing a hyperechoic septated mass affecting a major portion of the left hepatic lobe

Her initial laboratory results showed blood glucose, bilirubin, creatinine, total protein, and albumin without pathological changes. Alanine aminotransferase was increased to 97 IU/L (normal range (NR): <52), aspartate aminotransferase to 42 IU/L (NR: <39), gamma-glutamyl transpeptidase to 402 IU/L (NR: <64), alkaline phosphatase to 299 IU/L (NR: <299), lactate dehydrogenase to 455 IU/L (NR: <271), and C-reactive protein to 3.582 mg/dL (NR: <3). Moreover, the R factor was 0.6. Due to the characteristics and rapid worsening of her disease, she was referred to our service for an endoscopic ultrasound as part of the initial diagnostic approach. A transgastric endoscopic ultrasound was performed in which transpyloric examination revealed a hyperechoic, septated parenchymal image affecting a major portion of the left hepatic lobe, which was hypovascular on the Doppler ultrasound application (Figure 2).

**Figure 3:**
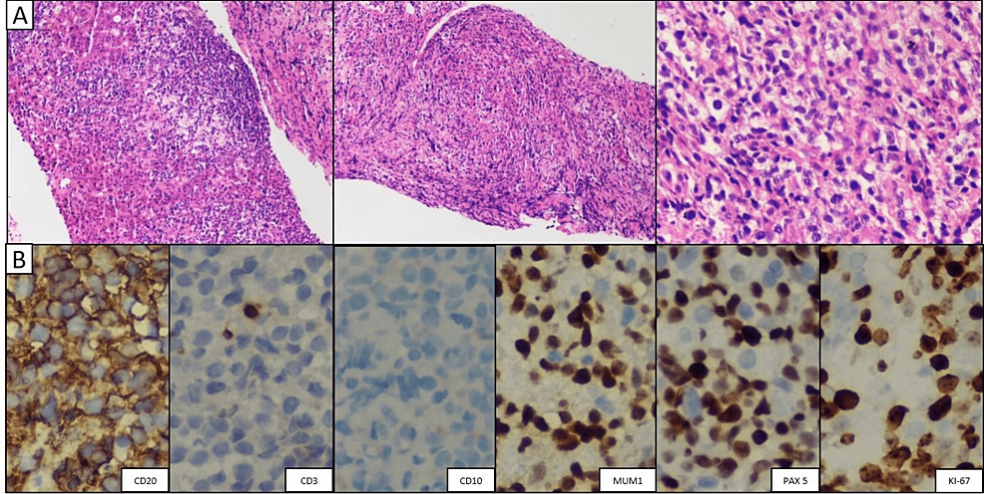
Fine needle aspiration liver biopsy a) Large centroblastic cells in a diffuse and pseudogranulomatous pattern. b) Non-germinal center diffuse large B-cell lymphoma immunohistochemistry panel

Using a 22-gauge needle (Acquire™ from Boston Scientific (Marlborough, Massachusetts, United States)), we performed a fine needle biopsy of the described lesion. The results of the biopsy resulted in a diffuse large B-cell lymphoma (DLBCL), and immunohistochemical markers CD20 and Ki67 were positive (Figure 3). We referred the patient to a tertiary referral center, where she received multiple rounds of chemotherapy with apparently good evolution.

## Discussion

Liver tumors are the fourth most common cause of cancer [1]. Malignant liver tumors can be divided into two groups: primary liver tumors which are tumors that have their origin in the liver and secondary liver tumors which are caused by dissemination from a primary neoplasia (Table 1).

**Table 1:** The most common types of primary and secondary liver lesions PHL: primary hepatic lymphoma

Primary liver lesions		Secondary liver lesions (origin)	
Hepatocellular carcinoma	70%	Pancreatic cancer	30-40%
Cholangiocarcinoma	15%	Colorectal cancer	30-50%
Combined hepatocellular carcinoma and cholangiocarcinoma	6%	Neuroendocrine tumor	20-46%
Angiosarcoma	2%	Small cell lung cancer	17%
Hemangioendothelioma	1%	Cutaneous melanoma	10-20%
PHL	<1%	Breast cancer	6-38%
		Gastric cancer	5-40%
		Non-small cell lung cancer	4%

The most common types of malignant liver tumors are detailed in Table 2.

**Table 2:** Common types of malignant liver tumors NAFLD: non-alcoholic fatty liver disease

Cancer type	Risk factors	Percentage rate	Survival rate
Hepatocellular carcinoma	Chronic hepatitis B or C, cirrhosis, alcohol abuse, NAFLD	75-85%	5-year survival rate 18%
Intrahepatic cholangiocarcinoma	Hepatitis B or C infection, primary sclerosing cholangitis, liver fluke infection, bile duct cysts, chronic biliary inflammation	15%	5-year survival rate 5-10%
Hepatic angiosarcoma	Exposure to vinyl chloride, thorium dioxide, arsenic, and radiation therapy; hemochromatosis; hepatitis C	2%	Median survival of 6 months
Hemangioendothelioma	Congenital trauma, radiation exposure	<1%	5-year survival rate 85-95%
Hepatic primary lymphoma	Immunodeficiency, autoimmune disease, chronic hepatitis B or C, Epstein-Barr virus	<1%	5-year survival rate 30-50%

Hepatocellular carcinoma (HCC)

HCC is the most common solid liver tumor and accounts for about 80% of all liver cancers [1-6]. The risk factors for HCC include chronic infection with hepatitis B or C, alcoholic liver disease, non-alcoholic fatty liver disease, and exposure to aflatoxins. Treatment options for HCC depend on the stage and severity of the disease and include surgical resection, liver transplantation, ablation therapy, transarterial chemoembolization, systemic therapy, and supportive care. Multidisciplinary management is recommended for HCC patients to optimize treatment outcomes [6-7].

Cholangiocarcinoma (CCA)

Depending on their anatomical site of origin, CCAs are classified into intrahepatic CCA (iCCA), perihilar CCA (pCCA), and distal CCA (dCCA). CCA is less common than HCC but still accounts for about 15% of all liver cancers [1]. It is slightly more common in males and has a peak incidence in between the fifth and seventh decades of life [8]. The risk factors for ICC include chronic liver disease, hepatitis B or C infection, primary sclerosing cholangitis, and exposure to toxins such as thorium dioxide. Surgery, when feasible, is the mainstay of treatment, but palliative treatments such as stenting, chemotherapy, and radiation therapy can also be used to improve quality of life and prolong survival in advanced cases.

Angiosarcoma

Hepatic angiosarcoma accounts for less than 2% of all liver cancers and although rare is considered the third most common primary liver tumor [9]. It is more common in men with a 3:1 ratio and is most frequently diagnosed in people aged 60 years or older [1,9]. Symptoms of hepatic angiosarcoma can include abdominal pain, fatigue, weight loss, loss of appetite, and a feeling of fullness in the abdomen. It occurs in association with exposure to known chemical carcinogens such as vinyl chloride monomer, thorotrast, anabolic steroids, and arsenic [9]. However, in 75% of cases, the etiology is not known [10]. Hepatic angiosarcoma is associated with high invasiveness, easy recurrence and metastasis, poor prognosis, and a short survival time, generally between six months [1].

Hemangioendothelioma

Hepatic hemangioendothelioma (HEHE) is a rare tumor of the liver that arises from the endothelial cells lining the blood vessels within the liver. It is considered a vascular neoplasm and is classified as an intermediate vascular tumor. The incidence of HEHE is estimated to be approximately one in 1,000,000 individuals, less than 1% of primary liver neoplasias [1]. Females have a higher incidence with the male-to-female ratio being 2:3 [11]. Currently, no etiology of HEHE has been identified yet, and it could be associated with oral contraceptives, alcohol exposure, and viral hepatitis [12]. Currently, there are no standardized guidelines for the management. The treatment options are broad and include chemotherapy, ablation, surgery, and liver transplantation, with inconsistent results [13].

Hepatic DLBCL

Hepatic DLBCL is a rare and aggressive subtype of non-Hodgkin lymphoma that originates in the liver accounting for only 0.016% of all non-Hodgkin lymphoma cases [4]. The disease is more common in males than females and usually affects individuals over the age of 60. The exact cause of DLBCL is unknown, but it is believed to be linked to immune dysfunction, genetic mutations, and chronic inflammation; multiple genes have been associated with the development of DLBCL. Symptoms of DLBCL of the liver may include abdominal pain, weight loss, fatigue, and loss of appetite [3]. However, many individuals with DLBCL of the liver may not have any symptoms until the disease has progressed. Diagnosis of DLBCL of the liver involves a combination of imaging tests, such as computed tomography (CT) scans, magnetic resonance imaging (MRI), and ultrasound. Liver biopsy is considered the gold standard in the diagnosis of primary hepatic lymphoma [14], but we consider endoscopic ultrasound-guided biopsies as the preferred option for the diagnosis. Treatment for DLBCL of the liver usually involves a combination of chemotherapy, immunotherapy, and radiation therapy. Surgery is considered the first line of treatment to remove the affected part of the liver. The traditionally used chemotherapy regimen is CHOP (cyclophosphamide, doxorubicin, vincristine, and prednisone) combined with Rituxan, which has shown good results [15]. The five-year survival rate for DLBCL of the liver is around 40%, but this can vary widely depending on the individual case [3].

## Conclusions

Hepatic DLBCL is a rare type of lymphoma that arises in the liver. Symptoms may include abdominal pain, weight loss, fatigue, and loss of appetite. Diagnosis involves a combination of imaging tests and biopsy. Treatment usually involves chemotherapy, immunotherapy, and radiation therapy, and prognosis depends on the stage of the disease and the patient's overall health. Further research is needed to better understand the causes of DLBCL of the liver and to develop more effective treatments for this rare and complex disease. Although rare, considering it part of the differential diagnosis for primary liver neoplasia is utterly important for the correct clinical practice.
